# Characterization of Endophytic Fungi from *Acer ginnala* Maxim. in an Artificial Plantation: Media Effect and Tissue-Dependent Variation

**DOI:** 10.1371/journal.pone.0046785

**Published:** 2012-10-08

**Authors:** Fenghui Qi, Tianzhong Jing, Yaguang Zhan

**Affiliations:** 1 School of Life Sciences, Northeast Forestry University, Harbin, China; 2 School of Forestry, Northeast Forestry University, Harbin, China; Centro de Investigación y de Estudios Avanzados, Mexico

## Abstract

The community of endophytic fungi associated with *Acer ginnala*, a common tree in northeastern China, was investigated. Four media, PDA, Czapek’s, WA and Sabouraud’s, were used to inoculate explants from seeds, annual twigs and perennial twigs (xylem and bark). Media strongly affected the isolated species number, but not colonization frequency (CF) or isolation frequency (IF). To investigate media effect further, a Principal Component Analysis (PCA) was done. As a result, two components accounted for 86.502% of the total variance were extracted. These two components were named as PDA-determined factor (accounted for 45.139% of the total variance) and Czapek’s-determined factor (accounted for 41.363% of the total variance), respectively. This result suggested that only two media, PDA and Czapek’s, could be used instead of all four media in this study without affecting the isolation results significantly. In total, ten taxa were isolated in this study. *Alternari*a sp., *Phomopsis* sp., *Neurospora* sp. and *Phoma* sp. were dominant endophytes while Pleosporales *Incertae Sedis* sp., *Cladosporium* sp., *Trichoderma* sp. and *Epicoccum* sp. were rare taxa. Different tissues/organs had different endophyte assemblages. All tissue/organ pairs had low Bray-Curtis indices (<0.3) except for bark and annual twigs (0.63). Compared to perennial twigs, annual twigs had a lower taxon number, lower isolate number, lower endophyte dominance and diversity indices. Seeds had distinct assemblage, lower similarity and similar low diversity indices to annual twigs. These results suggested that tissue type determines the endophyte assemblage while age determines the diversity.

## Introduction

Endophytic fungi, the symbionts that reside in the above ground tissues of plants, have been considered as a source of novel biologically active secondary metabolites [1]. These bioactive compounds include paclitaxel, podophyllotoxin, camptothecine, vinblastine, hypericin, and diosgenin, etc. (for review, see [2]). Fungal endophytes have profound ecological effects on plant. For example, they can affect the community structure and diversity of associated organisms (e.g. bacteria, nematodes and insects [3]), or affect the fitness and evolution of plant [4]. Recently studies have shown that environmental stochastic events (e.g. climatic factors) can cause the lifestyle of an endophyte to switch from beneficial/neutral to pathogenic [5], [6].

Fungal endophytes are omnipresent within plant, and Partida-Martinez & Heil proposed that there were no microbe-free plants in nature [7]. However, most of the endophytic fungi are yet to be discovered [8]. Furthermore, fungal endophytes were found in asymptomatic photosynthetic tissues of all major lineages of land plants, and the diversity, geographic distribution and host specificity of endophytes remain largely unknown [9]. There are two main techniques in endophytic fungal study, culture-dependent and culture-independent. Although cultivation-independent screening methods like direct PCR from plant tissue are used by an increasing number of researchers for diversity study [7], the cultivation-dependent isolating method is still important in the study of fungal endophytes, especially in isolation of bioactive compounds from endophytes [10]–[14]. In the cultivation-dependent isolation, the number of media is known to affect the number of isolated species. If the number of media increased, the isolation work is increased exponentially. So, is it possible to reduce the media number without significant sacrifice of isolation results? In a previous study, we identified some endophytic fungi rich in gallic acid from *Acer ginnala* Maxim., a tree mainly distributed in China and Korea [15]. In the present study we report on the diversity of endophytic fungi from the seeds and twigs of *A. ginnala*, comparing the effect of cultivation medium changes.

Organ and tissue specificity of fungal endophytes has been studied before. Limited tissue specificity has been showed in few plant (for instance, *Eucalyptus nitens* [16]) while the majority of previous studies showed obvious tissue specificity or little overlap [17]–[20]. As a result of adaptation to different physiological conditions in plants, different fungi dominate in distinctive tissues forming characteristic communities specific to each tissue type [21]. So, studying tissue specificity of fungal endophytes can help to discover the selective pressures occurring in a certain plant tissue type [21]. In this paper, we explore the endophyte community from different tissues of *A. ginnala*. Most studies of endophyte diversity have been done in wild environments. Perhaps the diversity in an artificial environment is different and lower. To test this, we selected a botanic garden to carry out our experiment.

## Materials and Methods

### Ethics Statement

No specific permits were required for the described field studies.

### Plant and Sample Locations

The plant materials were collected from Heilongjiang Forest Botanical Garden, a national forest park located in an urban district of Harbin city. The garden was 136 hectares in total area and more than 1,200 species of plants lived within it.

Materials were collected in late September of 2007. Seeds, annual twigs and perennial twigs were collected randomly from 20 trees. All samples were immediately brought to the laboratory in an icebox, and the tissues were screened for endophytic fungi within 2 days.

### Isolation and Identification of the Fungal Endophytes

Four media, PDA (diced potato 200 g, dextrose 15 g and agar 20 g) [22], water agar (WA), Sabouraud’s (glucose 40 g, peptone 10 g and agar 15 g) [23] and Czapek’s (sodium nitrate 2 g, potassium nitrate 1 g, potassium chloride 0.5 g, magnesium sulphate 0.5 g, ferrous sulphate 0.01 g, sucrose 30 g and agar 20 g) [24], [25], were used for inoculation. The endophytic fungi were preliminarily identified according to their microscopic characteristics, and then confirmed by their ITS-rDNA sequences. The methods for isolation and molecular identification of the fungal endophytes were adopted as presented in the previous study [15].

Colonization Frequency (CF) of an endophyte species was calculated as described by [26], which is equal to the number of segments colonized by a single endophyte divided by the total number of segments observed×100. Isolation Frequency (IF) was calculated as the number of isolates divided by the total number of segments observed×100. Relative frequency (RF) meant frequency of a given species divided by the sum of isolate frequencies of all endophytes×100. CFs and IFs were compared using SPSS 17.0 software with the chi-square test or Fisher’s exact probability test. Correspondence, regression and principal component analyses were also carried out using SPSS. After obtained the REGR factor score of each medium in each principal component (PC) in the principal component analysis (PCA), a total score of each medium was calculated to rank the media. The total score was the weighted average of REGR factor scores of each medium and the weight was the percentage of total variance each PC explained.

### Similarity and Diversity Analyses

To compare the similarity between tissues, a similarity index, Bray-Curtis, was calculated, and cluster analyses based on this index was also carried out. Species dominance, richness, evenness and diversity were calculated for the endophyte diversity analyses. Bootstrapping method was used to compare diversity indices, and consider there was a significant difference if the bootstrapping probability (BP) lowers 0.01. A modified *t* test was also used to compare Shannon’s index [27], [28]. All the diversity analyses and similarity analysis were done using PAST version 2.10 [29].

## Results

### Media Effect on Endophyte Isolation

**Figure 1:**
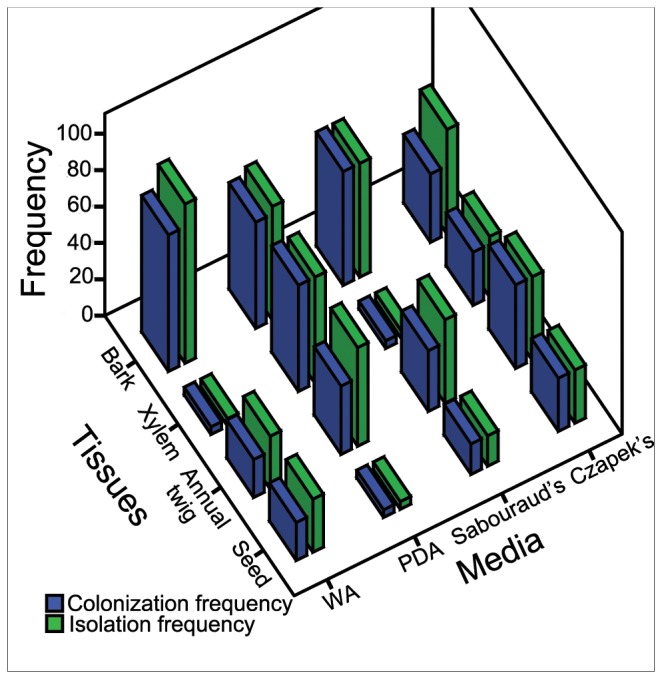
Colonization and isolation frequency of fungal endophytes from *A. ginnala.*

After epiphytic sterilization, 384 tissues were inoculated on four media and 145 endophytic isolates were obtained totally. The CF and IF of each medium are presented in Figure 1. Chi square test showed that medium had no significant effect on CF (χ^2^ = 3.023, *df* = 3, *P* = 0.388) and IF (χ^2^ = 3.313, *df* = 3, *P* = 0.346).

**Figure 2:**
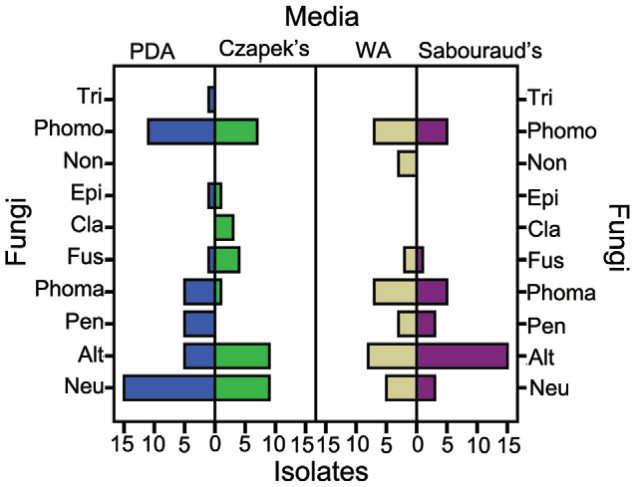
Media effect on species isolated from *A. ginnala.* Alt: *Alternaria* sp.; Pen: *Penicillium* sp.; Neu: *Neurospora* sp.; Cla: *Cladosporium* sp.; Phoma: *Phoma* sp.; Fus: *Fusarium* sp.; Phomo: *Phomopsis* sp.; Non: Pleosporales *Incertae Sedis* sp.; Tri: *Trichoderma* sp.; Epc: *Epicoccum* sp.

The effects of media on the endophyte taxon assemblage are shown in Figure 2. Among 10 taxa, only *Phomopsis* sp., *Fusarium* sp., *Phoma* sp., *Alternaria* sp. and *Neurospora* sp. were isolated on all four media; whereas *Trichoderma* sp. was only isolated on PDA, Pleosporales *Incertae Sedis* sp. only on WA, and *Cladosporium* sp. only on Czapek’s medium.

**Table 1:** Principal Component Analysis on the effect of media.

Component	Loading of each fungus										Total variance explained		
	Neu	Alt	Pen	Phoma	Fus	Cla	Epi	Non	Phomo	Tri	Total	%*	C %#
1	**0.930**	**−0.801**	0.656	0.105	−0.344	−0.247	0.637	−0.231	**0.981**	**0.982**	4.257	42.568	42.568
2	0.367	−0.186	**−0.738**	**−0.962**	**0.847**	**0.964**	**0.769**	−0.495	0.096	−0.076	3.918	39.182	81.750

Alt: *Alternaria* sp.; Pen: *Penicillium* sp.; Neu: *Neurospora* sp.; Cla: *Cladosporium* sp.; Phoma: *Phoma* sp.; Fus: *Fusarium* sp.; Phomo: *Pomopsis* sp.; Non: Pleosporales *Incertae Sedis* sp.; Tri: *Trichoderma* sp.; Epc: *Epicoccum* sp. Loadings over 0.7 were in bold.

* :percentage of variance.

# :Cumulative percentage.

**Figure 3:**
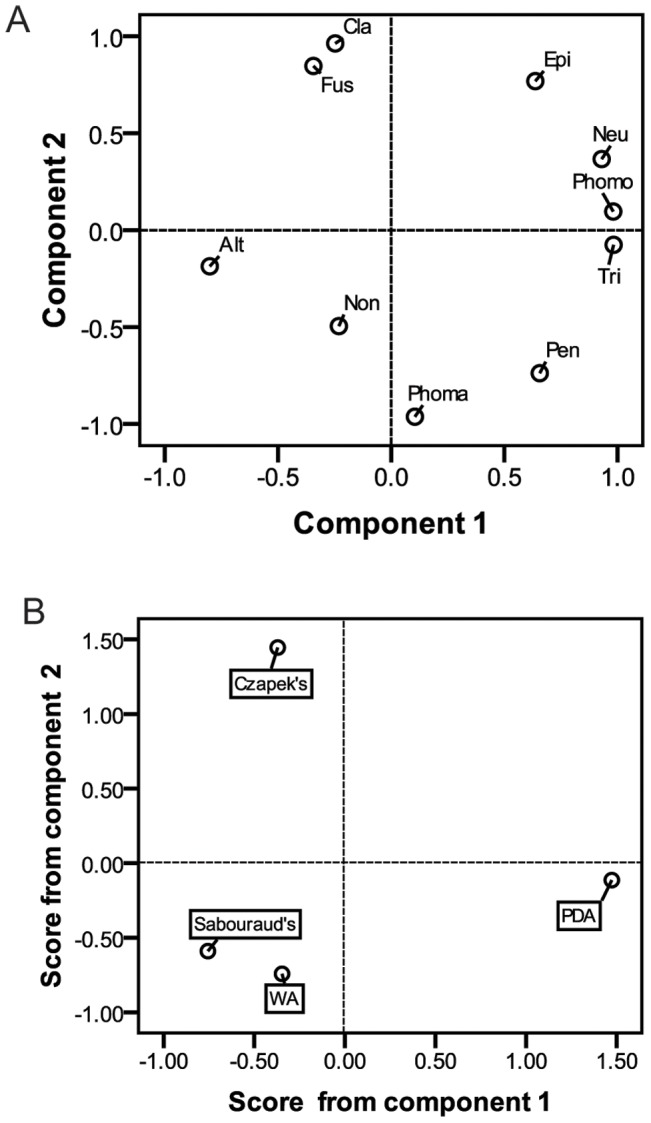
Principal component analysis of media effect on fungal endophytes isolation. A, loading plot; B, score plot. The legends see Figure 2.

**Table 2:** Relative frequency of fungal endophytes from *A. ginnala*.

Endophytes	Bark	Xylem	Perennial twig (Bark+ Xylem)	Annual twig	Seed	Total
*Phomopsis* sp.	13.10	0.69	13.79	5.52	1.38	20.69
Pleosporales *Incertae Sedis* sp.	–	–	–	–	2.07	2.07
*Cladosporium* sp.	–	0.69	0.69	–	1.38	2.07
*Fusarium* sp.	0.69	–	0.69	–	4.83	5.52
*Trichoderma* sp.	0.69	–	0.69	–	–	0.69
*Phoma* sp.	8.28	1.38	9.66	0.69	2.07	12.41
*Penicillium* sp.	3.45	–	3.45	2.76	1.38	7.59
*Alternaria* sp.	8.97	0.69	9.66	15.86	–	25.52
*Epicoccum* sp.	1.38	–	1.38	–	–	1.38
*Neurospora* sp.	4.83	13.10	17.93	4.14	–	22.07

Relative frequency (RF) means isolate frequency of a given endophyte divided by the sum of isolate frequencies of all endophytes×100.

**Figure 4:**
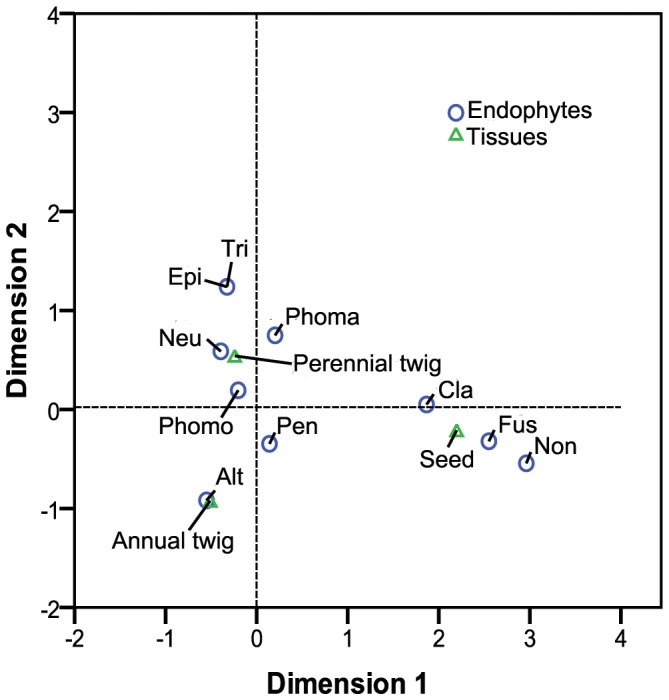
Correspondence analysis of endophytes and tissues. The legends see Figure 2.

**Figure 5:**
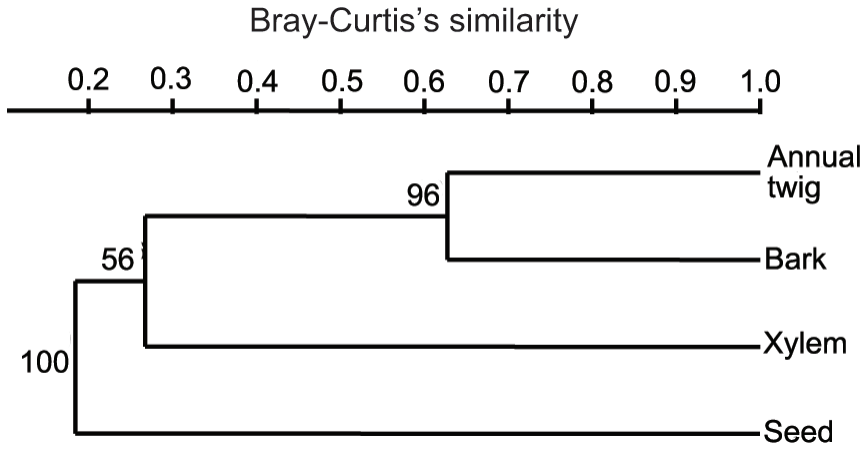
Clustal analysis of tissues. The dendrogram was drawn by PAST with Bray-Curtis’s similarity. The numbers on the branches were the support percentage from10000 bootstraps.

**Table 3:** α diversity indices and bootstrapping comparison between tissues.

Tissue	Taxa	Individuals	Simpson’s λ	Pielou’s J	Margalef	Shannon’s
Bark	8	60	0.2094	0.8295	1.71	1.725
Xylem	5	24	0.6389	0.4904	1.259	0.7893
Seed	6	19	0.2188	0.9274	1.698	1.662
Annual twig	5	42	0.3662	0.7683	1.07	1.237
Perennial twig	9	84	0.2126	0.7815	1.806	1.717
Whole tree	10	145	0.1819	0.8149	1.808	1.876
Annual twig-Perennial twig	0.029		0.001	0.855	0.055	0.003
Annual twig-Seed	0.779		0.069	0.057	0.076	0.102
Bark-Xylem	0.197		0.001	0.001	0.265	0.001

Whole tree represented by Bark + Xylem + Seed + Annual twig while perennial twig by Bark +Xylem.

To further investigate the effect of the media, A PCA was carried out and two components accounted for 86.502% of the total variance were extracted as a result (Table 1, Figure 3A). Therefore, the rest components could be omitted in further studies due to their trivial contribution to the total variance. Taxa mainly isolated on PDA (*Neurospora* sp., *Phomopsis* sp, *Trichoderma* sp.) or not (*Alternaria* sp.) had a high loading (>0.7) in PC1 while those mainly isolated on Czapek’s (*Fusarium* sp., *Cladosporium* sp. and *Epicoccum* sp.) or not (*Penicillium* sp. and *Phoma* sp.) had a high loading in PC2. PC1, therefore, could be named as PDA-determined factor, which accounted for 45.139% of the total variance and PC2 as Czapek’s-determined factor, which accounted for 41.363% of the total variance.

REGR factor scores from the first two PCs were used to draw score plot (Figure 3B). WA and Sabourand’s met together while PDA separated from them on PC1 and Czapek’s on PC2. Then the total score of each medium was calculated based on its REGR factor score. PDA had the highest total score (0.62), followed by Czapek’s (0), WA (−0.43) and Sabourand’s (−0.57).

### Endophyte Communtiy Variations between Tissues

58.3% of inoculators from bark colonized on the media while the percentages for xylem, annual twig and seed were 24.0%, 34.4% and 17.7%, respectively. And 62.5% of total isolates was from bark, 25.0% from xylem, 43.8% from annual twig and 19.8% from seed (Figure 1). Chi square test showed that there were significant differences among tissues, from the point of either CF (χ^2^ = 42.219, *df* = 3, *P*<0.001) or IF (χ^2^ = 46.306, *df* = 3, *P*<0.001).

Ten taxa of endophyte were identified from 145 isolates (Table 2). Correspondence analysis showed that there was obvious tissue tropism among the endophytes (Figure 4). Pleosporales *Incertae Sedis* sp., *Cladosporium* sp. and *Fusarium* sp. were mainly distributed within seed, *Alternaria* sp. tended to live in annual twigs, and the other taxa were prone to reside into perennial twig except for *Penicillium* sp. which showed nearly same tropism to the three organs. *Phomopsis* sp. and *Phoma* sp. inhabited all tested tissues, *Neurospora* sp. and *Alternaria* sp. did not live in seed.

### Endophyte Community Similartiy and Diversity Difference between Tissues

After the Bray-Curtis index was calculated, a cluster dendrogram was drawn (Figure 5). Annual twigs had a high similarity (>0.6) to the bark of perennial twigs while seeds had a low similarity (<0.2) to annual twigs.

The α diversity indices of each tissue type are shown in Table 3. In total, 60 isolates of 8 taxa from bark, 24 of 5 from xylem, 42 of 5 from annual twigs and 19 of 6 from seeds were collected. Bootstrapping comparison showed that annual twigs were significantly different from perennial twigs with respect to dominance and diversity (BP<0.01), but not in evenness and richness (BP>0.01); while seed was not distinct with annual twigs in terms of all four kinds of indices (BP>0.01), and bark was significantly different from xylem with all four indices (BP<0.01) except for richness (BP>0.01).

A series of *t* tests were also carried out to compare Shannon diversity between tissues. The results showed that there was significant difference between perennial twigs and annual twigs (*t* = −3.2165, *df* = 81.152, *P* = 0.0019), but no significant difference between seeds and annual twigs (*t* = 1.7725, d*f* = 44.957, *P* = 0.0831). The *t* test results were consistent with those of bootstrapping.

## Discussion

Four media were used to isolate endophytic fungi from *A. ginnala*. The results showed that there was no significant difference between media either from the point of colonization or isolation frequency (CF or IF; Figure 1). This meant that if the number of isolates were only considered, anyone of these four media should be used and more media should be not necessary. So the media effect should be concentrated on the effect of isolated species, i.e. the composition of endophyte community.

It was readily apparent from Figure 2 that media affected the composition of endophyte community, which meant that if more species of endophytes were desired, more types of media should be used. However, with every one medium added, the isolation work would be doubled. So, the wise strategy was to use fewer media combinations obtain an acceptable result without affecting the isolated taxa and their amount significantly. For this reason, a PCA was done and each medium was scored. The first two components, which were named PDA-determined factor and Czapek’s-determined factor, respectively, accounted for 86.502% of the total variance. Combining the loading plot, score plot and the total score of each medium, we concluded that with only the two media, PDA and Czapek’s, the isolation results would be acceptable for this study.

Ten taxa in total were isolated in this study. The ITS data and phylogenetic tree for identification were not shown since they have been published previously [15]. Compared with other studies, the number of isolated taxa in this study was lower. The endophyte diversity of the whole tree (represented by perennial twig, seed and annual twig) was also low. The Shannon-Wiener diversity index (H’) was only 1.808 (the H’ index is usually between 1.5 and 3.5). One reason to account for this is the lower number of sampled trees, which would significantly affect the number of isolated species [30]. Another reason was the number of sites, for significant differences in endophyte assemblages were detected in a global comparison of sites [31] and samples from different geographical origins were clearly separated [16]. Previous data have also shown that of 55 isolated fungal species, 20 species isolated only from 1 site, and only 12 species isolated from all five sites [30]. The type of tissues was perhaps the third reason for the lower diversity index. In this study only twigs and seeds were used resulting in just 10 taxa were recovered. There is plentiful data demonstrating that twigs have lower fungal endophytes than leaves. For example, only 15 taxa were recovered from the seed of *Salix fragilis* and *Quercus robur* [32], 26 from *Theobroma cacao* [33] and six from *Theobroma grandiflorum* [33]. In contrast, hundreds of endophytes were found in the leaves. For example, 242 morphotypes in the leaves of *Heisteria concinna* and 259 in *Ouratea lucens* were detected [34]. Also, twigs had less isolates (e.g. 134 vs. 1801 in *Quercus ilex* [35]) and endophytic colonization rate (e.g. 35.4% vs. 50.4% in *Lippia sidoides* [36]) than leaves. Compared to leaves, seeds are also shown to have less isolates. For example, only 16 endophytic isolates were obtained from 800 surface-sterilized seeds of western white pine *Pinus monticola* while 2003 fungal endophytes from 750 surface-sterilized needles [18]. The last reason may be related to the ecological environment of the site which was a botany garden. Endophytes increased in incidence, diversity, and host breadth from arctic to tropical sites [9], and the number of endophyte species in an urban plantation and a regenerated (managed) forest (80 year old) were only 30% and 67% of that in an old-growth forest (300 years old), respectively [37]. Although, there were few studies on a non-wild environment, we believe that the ecological environment is a vital factor to affect the diversity of fungal endophytes.

Of the ten taxa, *Alternaria* sp. was the most frequent (25.52%) while *Trichoderma* sp. was the rarest (0.69%). *Alternari*a sp., *Phomopsis* sp., *Neurospora* sp. and *Phoma* sp. were dominant endophytes, as each of them was present over 10% in RF; whereas another four taxa, Pleosporales *Incertae Sedis* sp., *Cladosporium* sp., *Trichoderma* sp. and *Epicoccum* sp. were rare taxa (less than 5% in RF).

In this study, it can be inferred from Figure 4 that *Alternaria* sp. was apt to inhabit the annual twigs, Pleosporales *Incertae Sedis* sp., *Cladosporium* sp. and *Fusarium* sp. had a preference of seeds, the rest taxa favored perennial twigs. There is ample evidence that fungal endophytes are tissue specific. For example, *Cladosporium tenuissimum* resides only within leaf of *Eucalyptus nitens*, *Phomopsis* sp. only within leaf, *Trichoderma harzianum* only within twigs subjected to the drying regime [16]; *Phoma* spp. only distributed withtin xylem of *Tripterygium wilfordii*, *Alternaria alternate* only within leaf [38]; *Phomopsis* spp. dominated the leaves of *Acer macrophyllum*, whereas *Diplorlinn ncerina* the twigs [39]. However, it is not necessary to compare the endophyte communities of the same tissues between distinct hosts, for host species, even closely related species [13], [40], has been shown the major factor shaping endophyte assemblages [21].

In this study, the type of tissue/organ had a statistically significant effect on colonization and isolation frequency. Bark CF and IF was larger than xylem, which was consistent with the findings in *Pinus tabulaeformis* [41]. The bark and xylem Bray-Curtis index is 0.26, which indicates a low similarity in endophyte assemblage. Table 3 shows that the α diversity difference lay in all four types of biodiversity indices except for richness. Bark had higher evenness and diversity, which was consistent with the usual findings that fungal diversity and abundance in xylem regions was lower than the bark [42].

Endophytes accumulate as tissue ages [9], [43], which have been demonstrated in leaf and needle [43]–[45], bark [45], xylem [45] and whole tree or whole forest [37]. In this study, nine taxa representing 87.5% of total isolates were isolated from perennial twig while only five taxa representing 43.8% of total isolates were from annual twigs. This also supports the age hypothesis presented at the beginning of this paragraph. However, the findings in *Pinus tabulaeformis* are contrary [41], perhaps the age difference was not big enough (one or two years difference). Aside from the lower number of taxa and isolates, annual twigs had statistically lower dominance and diversity indices (BP<0.01, Table 3). Since horizontal transmission of fungal endophytes is probably the rule for woody plants [16], these results suggest that this horizontal transmission is going on with age. Annual twigs and the bark of perennial twigs had a Bray-Curtis index of 0.63 and shared the same clad in Figure 5 with a bootstrap support of 96%. This result indicates that within annual twigs, the bark probably dominates the endophyte assemblage. This was not surveyed in this study.

In comparison to the abundance of data on leaf and stem endophytes, few reports are focused on seed endophytes. Like this study, these limited data showed that seeds had significantly fewer fungal endophyte taxa, lower diversity and different assemblage to other tissues/organs. For example, only 4–12 species were recovered from seven plant oil seeds [46]. In endophytes from the seed of seven oil plants, only one plant had a Shannon’s index over 2.5 [46], and only two with Simpsons indices over 0.5 [46]. The seed of coffee’s (*Coffea arabica*) diversity index (Fisher’s alpha) was far less than that of stem and leaf (3.6 vs. 17.0 and 31.1, respectively) [47]. The predominant endophytes isolated from seeds of *Pinus monticola* were of *Cladosporium s. lat.*, a contrast to needles in which enophytes belonging to Rhytismataceae dominated [18]. Although the seed and the annual twig grow out annually, both had lower diversity indices. In this study, the Bray-Curtis index was only 0.16, which indicated a low similarity in endophyte assemblage. These results suggested that tissue type determines the endophyte assemblage while the age determines the diversity, which has also been demonstrated in leaves [43], [44].
